# Construction of a Neoantigen Prognostic Model for Gastric Adenocarcinoma Based on Multi-Omics Data Mining and the Design of mRNA Vaccines and Targeted Drugs

**DOI:** 10.3390/ijms27177712

**Published:** 2026-08-28

**Authors:** Jiaxiang Liang, Zhipeng Xie, Yingjie Sun, Yuheng Tang, Samina Gul, Qi Qi, Jianyu Pang, Yongzhi Chen, Hui Wang, Jiehui Zhang, Wenru Tang, Xuhong Zhou

**Affiliations:** 1 Tumor and Aging Laboratory, Kunming University of Science and Technology, Kunming 650500, China; liangjiaxiang2001@163.com (J.L.); 13977391933@163.com (Z.X.);; 2 School of Integrative Medicine, Nanjing University of Chinese Medicine, Nanjing 210023, China; 3 Office of Science and Technology, Yunnan University of Chinese Medicine, Kunming 650500, China

**Keywords:** GAC, single-cell analysis, copy number variation (CNV), single nucleotide variation (SNV), cancer immune-related genes, machine learning, neoantigen-related genes, mRNA template, TME, molecular docking, MD

## Abstract

This study systematically explored immune targets in gastric adenocarcinoma (GAC) suitable for mRNA vaccine development. Based on multi-omics data from public databases, we first screened a set of potential tumor-associated antigen genes. Subsequently, using ten machine learning algorithms, we constructed 101 prognostic models and, through optimization and comparison, selected the Random Survival Forest (RSF) method to establish a clinical prognostic model for GAC consisting of seven genes (TYMP, IFGN, ITGAX, GBP5, GBP4, STAT1, CD84). At both the genetic and protein levels, these genes were closely associated with the antigen presentation process, suggesting the potential functional role of this model in antigen presentation. Further analysis of the immune infiltration characteristics in GAC preliminarily revealed its possible immune evasion mechanisms. Building on this, we designed candidate mRNA vaccine templates for GAC using the mRNAdesigner platform. Additionally, this study investigated the potential roles of the above seven genes in GAC progression and screened small-molecule compounds targeting these genes. Molecular dynamics simulations (MD) were performed to verify the binding stability between these compounds and their corresponding proteins. This study comprehensively simulated the tumor microenvironment (TME) and antigen presentation process in GAC, evaluated the clinical translation potential of the neoantigen prognostic model and its predictive value for immunotherapy, and provided a preliminary design scheme for an mRNA vaccine against GAC. The findings offer new evidence for identifying immune therapy targets in GAC and are expected to advance the development of immunotherapy strategies for GAC.

## 1. Introduction

Gastric cancer (GC) is one of the most common malignancies of the human digestive system and the fifth most frequently diagnosed cancer worldwide [1]. The incidence of GC is particularly high in Asia, Latin America, and parts of Central and Eastern Europe, whereas in North America and most Western European countries, it is no longer a common cancer [2]. Chronic inflammation induced by gut microbiota, dietary habits, smoking, alcohol consumption, obesity, and other factors is a key contributor to the development of GC [3]. GAC is the most common pathological type of GC, accounting for over 95% of all cases. Radical resection followed by adjuvant therapy is the standard treatment for GAC [4]. However, with conventional treatment, the 5-year survival rate is approximately 50%, and about 70% of patients experience recurrence or metastasis within 5 years [5]. Research on GAC in the field of immunotherapy remains relatively limited. Therefore, developing more diversified treatment approaches is crucial for improving outcomes in GAC patients.

In recent years, the paradigm of cancer treatment has gradually expanded from traditional approaches such as surgery, chemotherapy, and radiotherapy to the novel field of immunotherapy [6]. As a pivotal branch of immunotherapy, cancer vaccines aim to deliver tumor antigens to the body, thereby activating and amplifying host-specific T-cell immune responses to achieve precise elimination of tumor cells [7]. Compared with other immunotherapies, cancer vaccines offer potential advantages such as low toxicity, high specificity, and durable immune memory, demonstrating broad application prospects in various solid tumors [8,9]. In the field of GC, dendritic cell (DC) vaccines have demonstrated promising potential in early clinical trials for advanced GC by efficiently presenting antigens and activating T cells [10]. These explorations have laid the foundation for immunotherapy in GC while also highlighting the need to develop more efficient and safer vaccine platforms. Among various vaccine technologies, mRNA vaccines have gradually become a research focus in tumor immunotherapy due to their unique advantages [8]. Compared with traditional vaccines, mRNA vaccines carry no risk of infection and cannot integrate into the host genome, fundamentally avoiding the safety concern of insertional mutations [11]. Furthermore, mRNA vaccines can be rapidly and mass-produced through in vitro transcription, with low production costs and flexible processes, making them particularly suitable for the development of personalized cancer vaccines [8,9]. Through sequence optimization and improvements in delivery systems, the stability and immunogenicity of mRNA vaccines have been significantly enhanced, enabling the induction of robust T-cell responses. This has been preliminarily validated in clinical studies of tumors such as lung cancer and prostate cancer [12,13]. Tumor-specific neoantigens derived from somatic mutations in cancer cells serve as ideal targets for mRNA vaccine design [14]. However, research on mRNA vaccines for GC remains in its early stages. Therefore, this study primarily focuses on characterizing the tumor microenvironment of GC and, based on these findings, developing mRNA vaccines targeting neoantigens.

In this study, we aimed to construct and validate a neoantigen-based prognostic gene model for GAC, with the goal of improving patient prognosis and informing therapeutic strategies. First, we integrated single-cell RNA sequencing (scRNA-seq) datasets from the GEO and Omix databases to enhance the breadth of our data. Based on the scRNA-seq data, we accurately identified the gastric adenomatous epithelial cell clusters within GAC samples and defined them as cancer cell populations through CNV analysis. Subsequently, we integrated somatic SNV data from the TCGA database with cancer immune-related gene sets obtained from the ImmPort database, and performed intersection analysis with genes upregulated in GAC. Building upon this, we employed a machine learning framework comprising 101 algorithm combinations for screening and ultimately selected RSF method within the optimal model range to construct a neoantigen-related prognostic model for GAC. To explore the association between this model and immune response mechanisms, we further analyzed the correlation between prognostic genes and antigen-presenting cells (APCs) and validated the functional interactions between these genes and MHC II molecules through a protein–protein interaction network. Additionally, by analyzing the characteristics of TME in GAC, we preliminarily revealed potential immune evasion mechanisms in GAC. Finally, this study investigated the functional mechanisms of key genes in the prognostic model and screened small-molecule compounds capable of targeting and regulating these genes. Through molecular docking and MD, we preliminarily verified the feasibility of binding between these candidate drugs and their target proteins, thereby providing a theoretical basis for exploring combined therapeutic strategies for GAC.

**Statement of Significance**

Problem or Issue: Gastric adenocarcinoma (GAC) is a highly heterogeneous malignancy with poor prognosis, yet reliable biomarkers for risk stratification and effective immunotherapeutic strategies remain limited.

What is Already Known: Neoantigens derived from somatic mutations serve as ideal targets for cancer vaccines, and multi-omics data offer opportunities for precision oncology. However, integrative frameworks that link neoantigen-related genes to prognostic modeling, vaccine design, and targeted drug discovery are lacking.

What this Paper Adds: This study establishes a multi-omics data mining pipeline integrating machine learning to construct a seven-gene neoantigen-related prognostic model (TYMP, IFNG, ITGAX, GBP5, GBP4, STAT1, CD84) for GAC. It further translates this model into rationally designed mRNA vaccine candidates and identifies potential targeted compounds validated by molecular dynamics simulations.

Who would benefit: Clinicians and oncologists can use this model for patient risk stratification; computational biologists and pharmacologists may leverage the identified targets for vaccine and drug development; patients may benefit from personalized immunotherapy strategies.

## 2. Results

### 2.1. Single-Cell Landscapes and Gastric Gland Epithelial Cells/Cancer Cells

**Figure 1:**
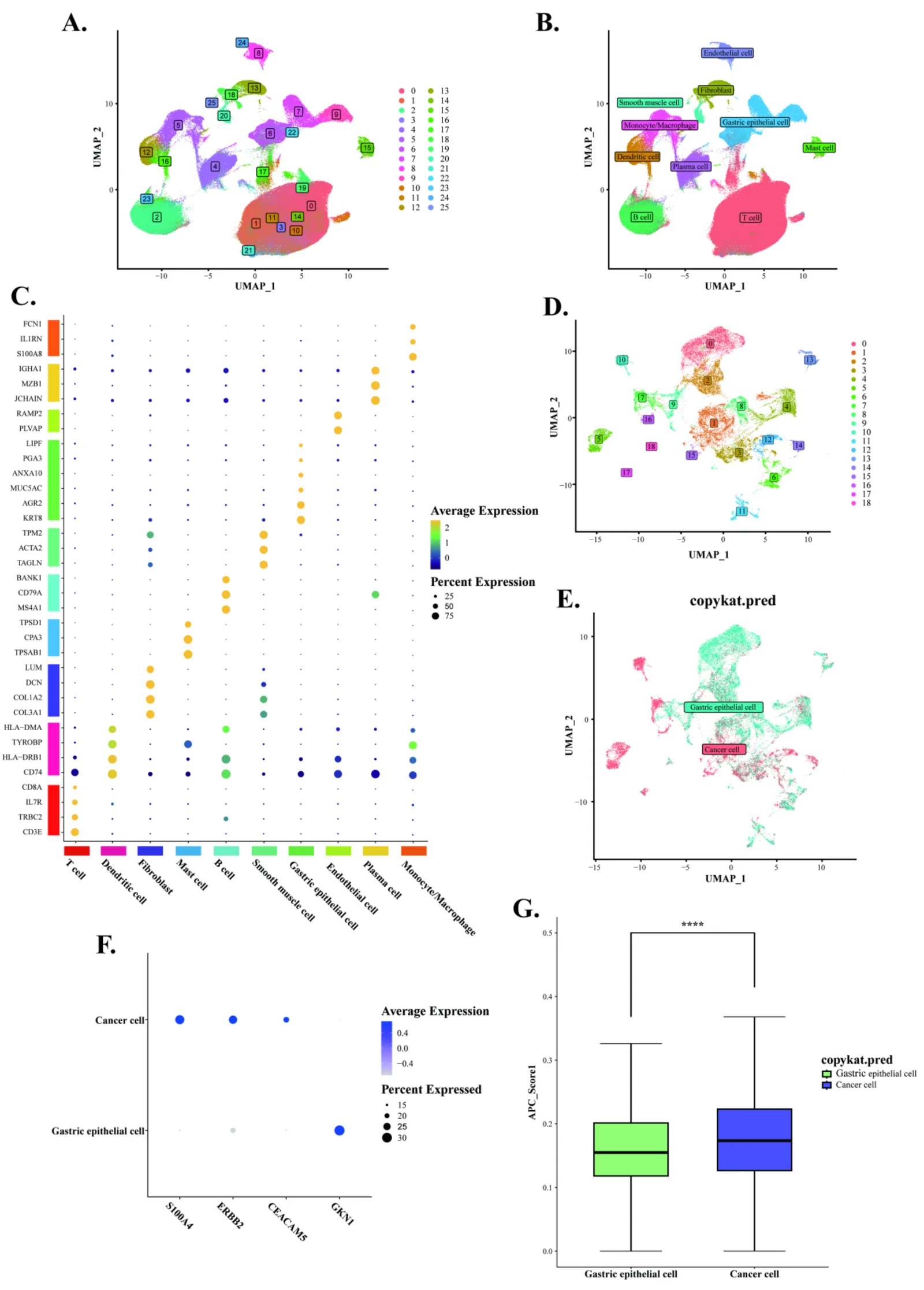
Single-cell transcriptomic atlas reveals cellular heterogeneity and CopyKAT identifies malignant cells. (**A**) Dimensionality reduction and clustering identified 25 cell clusters. (**B**) Marker gene annotation identified 10 cell types. (**C**) Heatmap showing the expression levels of specific genes in each cell type. (**D**) The CopyKAT algorithm further divided gastric gland epithelial cells into 18 subclusters. (**E**) The CopyKAT algorithm identified cells with chromosomal aneuploidy features as malignant clusters. (**F**) Expression differences in gastric cancer-related marker genes between normal cells and cancer cells. (**G**) Differences in antigen presentation scores between cancer cell clusters and normal cell clusters. (* *p* < 0.05; ** *p* < 0.01; *** *p* < 0.001; **** *p* < 0.0001).

The technical workflow of this study is illustrated in Figure S1. First, batch effects were removed from the merged GAC single-cell samples (Figure S2A,B), followed by QC (Figure S2C,D), resulting in a final selection of 273,924 high-quality cells. Subsequently, these cells were grouped into a total of 25 clusters (Figure 1A). Through automated and manual annotation, 10 distinct cell subpopulations were identified, including gastric gland epithelial cells, endothelial cells, fibroblasts, smooth muscle cells, T cells, B cells, plasma cells, mast cells, monocyte-macrophages, and DCs (Figure 1B). Bubble plots were used to visualize marker gene expression within each cell cluster (Figure 1C).

To identify cancer cells within the gastric gland epithelial cell population, we applied the CopyKAT algorithm and defined 3232 aneuploid gastric gland epithelial cells as cancer cells (Figure 1D,E). We visualized the expression of S100A4, ERBB2, CEACAM5, and GKN1 in the two cell populations using bubble plots to validate the accuracy of cell clustering (Figure 1F), and additionally generated heatmaps depicting gene expression per cell sample for both groups (Figure S3). Subsequently, pathway scoring was performed on gastric gland epithelial cells and cancer cells. We found that the antigen presentation score in the cancer cell population was significantly higher than that in the gastric adenomatous epithelial cell population (Figure 1G). These findings suggest that the antigen presentation signaling pathway in GAC patients may be influenced by gastric adenomatous epithelial cells and cancer cells.

### 2.2. Construction of Differentiation Trajectories for GAC Cancer Cells and SNV Analysis in GAC

**Figure 2:**
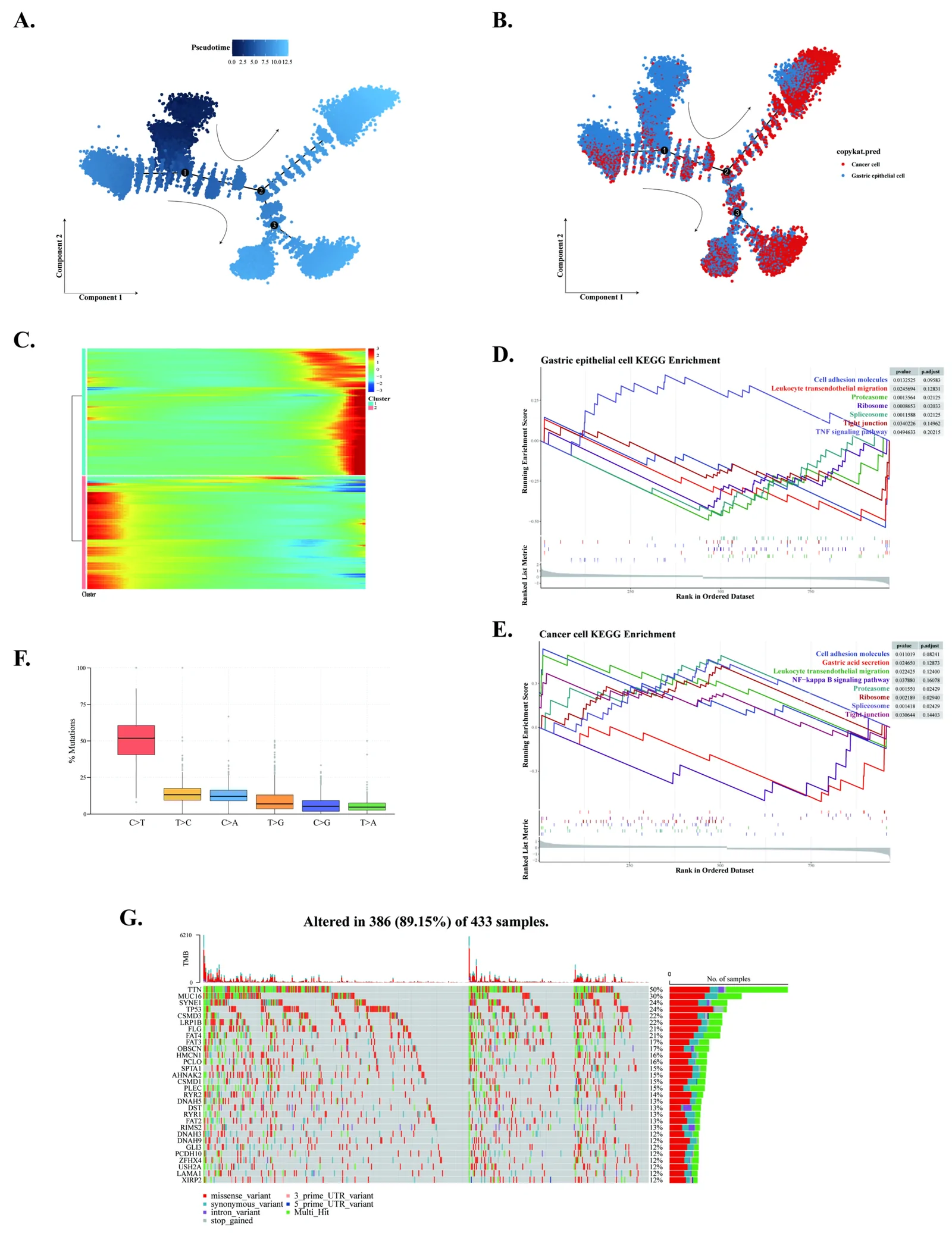
Analysis of Malignant Transformation Trajectory and Genomic Variation Characteristics of Gastric Gland Epithelial Cells. (**A**) Pseudotime analysis reconstructs the malignant transformation trajectory of gastric gland epithelial cells. (**B**) Distribution of gastric gland epithelial cells and cancer cells in pseudotime analysis. Numbers 1, 2 and 3 are labels for branch nodes on the pseudotime trajectory and have no biological significance. (**C**) Pseudotime heatmap displaying the dynamic expression changes in genes associated with gastric gland epithelial cells and cancer cells. (**D**,**E**) GSVA pathway activity analysis of gastric gland epithelial cells and cancer cells. (**F**) Mutation types and mutation frequencies in GAC. (**G**) Top 30 SNV genes in GAC.

We used Monocle to construct the growth and differentiation trajectories of gastric gland epithelial cells and cancer cells in GAC (Figure 2A). The results showed that epithelial cells were predominantly located in the early differentiation stage, while cancer cells clustered in the late stage (Figure 2B). A differentiation heatmap illustrating the expression patterns of marker genes across different differentiation stages further confirmed the validity of the two cell group classifications (Figure 2C). Based on differentiation genes associated with epithelial and cancer cells, we performed GSEA. The results revealed that the TNF signaling pathway was significantly upregulated in gastric gland epithelial cells, while other pathways showed a downward trend (Figure 2D). In contrast, multiple cancer-related pathways were markedly upregulated in cancer cells (Figure 2E). These findings indicate that the transformation from gastric adenomatous epithelial cells to cancer cells is a continuous differentiation process accompanied by dynamic, multi-stage gene expression evolution. During this process, gastric adenomatous epithelial cells often reside in an inflammatory microenvironment characterized by TNF pathway activation, which may progressively drive their transition toward a malignant phenotype, ultimately leading to significant enrichment of pro-oncogenic pathways in the cancer cell stage.

During the analysis of SNVs in GAC, we observed that C > T mutations were the most frequent type (Figure 2F). Based on SNV data from the TCGA database, we further visualized the top 30 genes with the highest mutation frequencies in GAC patients, among which the TNN gene exhibited a mutation rate as high as 50% (Figure 2G).

### 2.3. Screening and Construction of a GAC Neoantigen Prognostic Model Using 10 Algorithm Combinations

**Figure 3:**
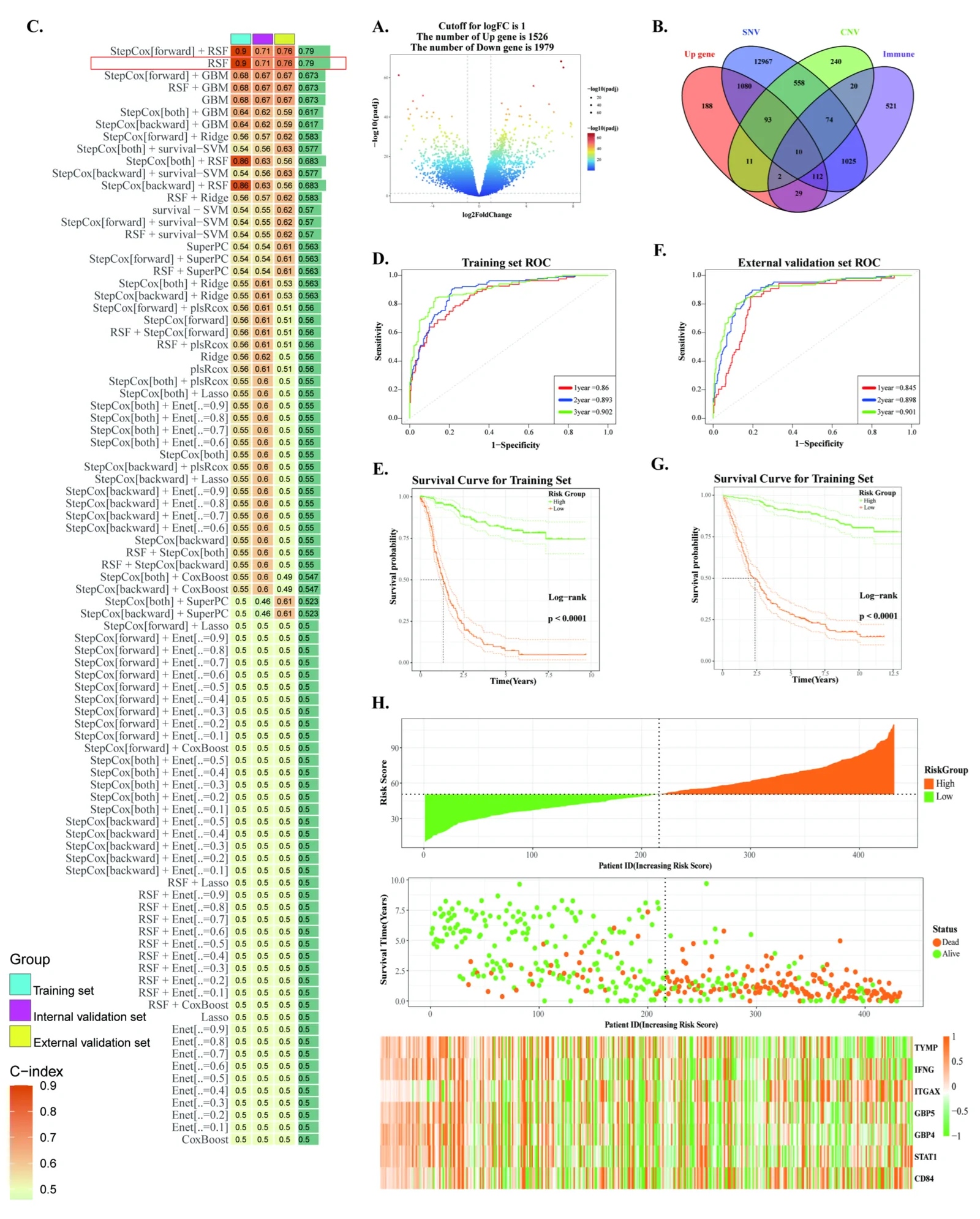
Construction and Validation of the Predictive Risk Model. (**A**) Volcano plot of differential expression analysis in GAC. (**B**) Intersection of upregulated genes in GAC, key genes in the carcinogenesis trajectory, SNV genes, and cancer-related immune genes. (**C**) Screening of prognostic risk models using 101 machine learning algorithms. (**D**) ROC curve of the training cohort. (**E**) KM curve of the training cohort. (**F**) ROC curve of the external validation cohort. (**G**) KM curve of the external validation cohort. (**H**) Survival scatter plot of the training cohort.

We integrated bulk RNA-seq data for GAC (Figure S4A,B). To identify candidate genes suitable for neoantigen mRNA vaccine design, differential expression analysis was performed between tumor tissues and adjacent normal tissues based on the training dataset, leading to the identification of 1526 upregulated genes (Figure 3A), defined as GAC overexpressed genes. Subsequently, these overexpressed genes were intersected with CNV genes derived from single-cell data analysis, SNV genes from GAC, and cancer-related immune genes, ultimately resulting in the selection of 10 key genes for constructing the GAC neoantigen prognostic risk model (Figure 3B).

We employed ten machine learning methods to generate 101 algorithmic combinations for screening the optimal modeling approach. By analyzing the C-index of each machine learning combination in the training, internal validation, and external validation sets, we ultimately selected RSF to construct the prognostic model. The prognostic genes identified were TYMP, IFNG, ITGAX, GBP5, GBP4, STAT1, and CD84 (Figure 3C and Figure S4C). Subsequently, we found that the GAC neoantigen prognostic model achieved AUC values around 0.85 for 1-year, 3-year, and 5-year survival in the training, internal validation, and external validation sets (Figure 3D,F and Figure S4D). Based on the median neoantigen risk score, patients were stratified into high-risk and low-risk groups. The results showed that overall survival (OS) was significantly lower in the high-risk group compared to the low-risk group (Figure 3E,G and Figure S4E). Furthermore, the low-risk group contained a higher number of surviving patients, while the high-risk group had a greater number of deceased patients (Figure 3H and Figure S4F,G). These results demonstrate that the GAC neoantigen prognostic risk model exhibits strong predictive performance.

### 2.4. Correlation Analysis Between Prognostic Genes and Antigen Presentation Response

**Figure 4:**
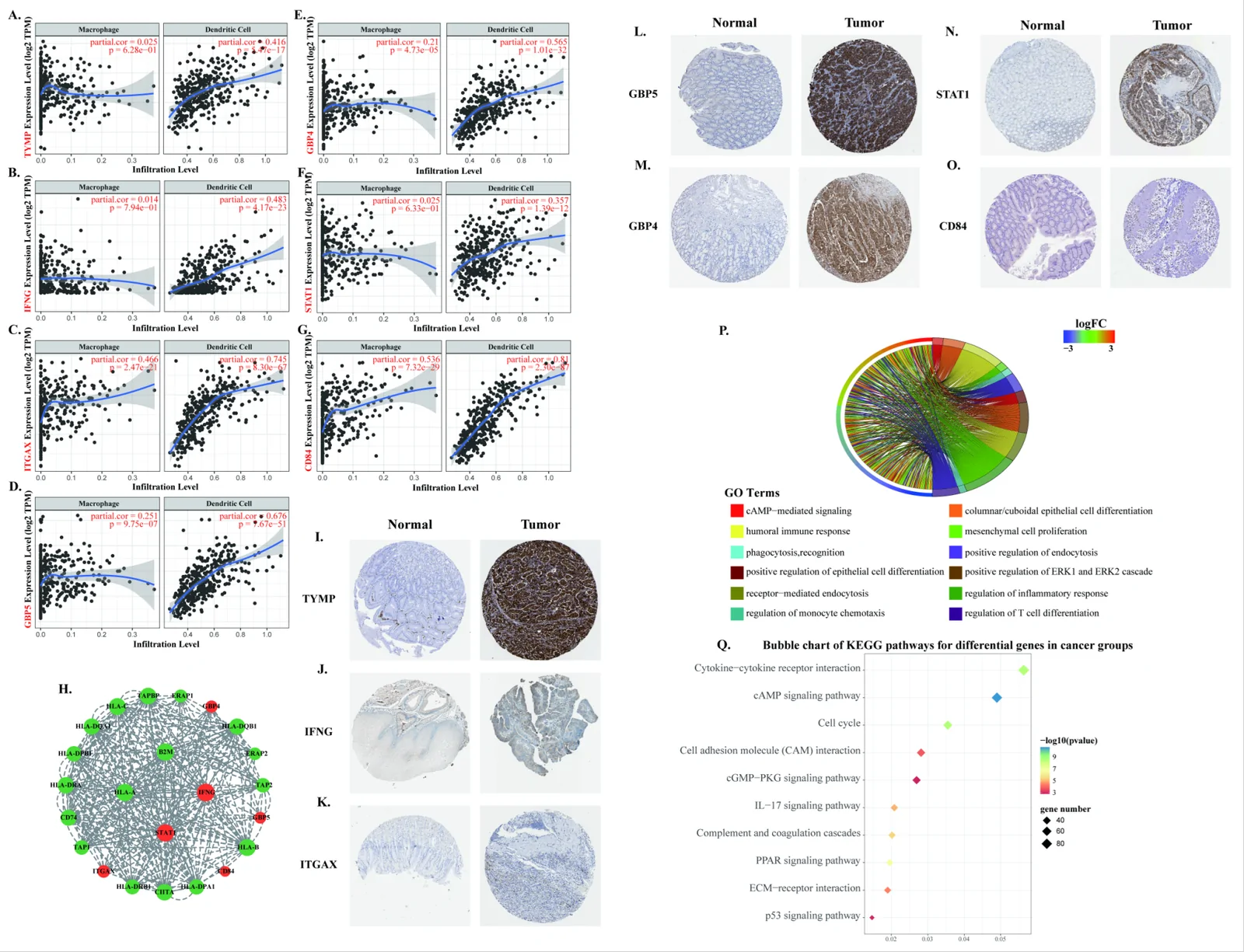
Antigen Presentation Effect of the GAC Prognosis Model and Pathway Performance in GAC. (**A**–**G**) Correlation between GAC prognosis-related genes and immune infiltration of macrophages and DCs. (**H**) Protein–protein interaction network diagram of prognosis-related genes and antigen presentation-related genes. (**I**–**O**) Pathological section images of prognosis-related genes in normal tissues and cancer tissues. Data were from Human Protein Atlas database (HPA, https://www.proteinatlas.org/, accessed on 25 August 2026). (**P**) GO pathway analysis of GAC. (**Q**) KEGG pathway analysis of GAC.

We performed correlation analysis between the selected potential antigen genes and the primary antigen-presenting cells (macrophages and DCs). The results showed that all seven genes exhibited significant positive correlations with both cell types (Figure 4A–G). Further analysis of the interaction between antigen genes and MHC-related genes via a protein–protein interaction (PPI) network revealed that IFNG and STAT1 were closely linked to MHC-related genes, while ITGAX, GBP5, GBP4, and CD84 also showed certain interactions with MHC-related genes. However, no direct interaction was observed between the TYMP protein and MHC-related proteins (Figure 4H). The above results indicate that most prognostic genes are closely associated with antigen presentation function. Among them, IFNG and STAT1 may play a key regulatory role in this process, and the set of prognostic genes may cooperatively regulate antigen presentation, collectively influencing tumor immune responses.

Additionally, we obtained immunohistochemical images of TYMP, IFNG, ITGAX, GBP5, GBP4, STAT1, and CD84 in cancerous and normal tissues from the HPA database (Figure 4I–O). The results showed that the expression levels of these proteins were significantly higher in tumor tissues compared to normal tissues, and they produced translatable protein products, meeting the criteria for mRNA vaccine targets. Based on this, we designed candidate mRNA vaccine sequences for each of these seven genes using the mRNAdesigner platform (Supplementary Data).

GO enrichment analysis showed that significantly enriched pathways in GAC patients primarily involved TME, tumor cell proliferation, and macrophage recognition and phagocytosis processes (Figure 4P). KEGG analysis further revealed core signaling pathways participating in these biological processes, including the PI3K-Akt signaling pathway, MAPK signaling pathway, and cytokine–cytokine receptor interaction pathway (Figure 4Q). These results suggest that the aforementioned prognostic genes not only exhibit individual correlations with APCs but may also function as a coordinated module, collectively regulating immune recognition and inflammatory response networks in GAC.

### 2.5. The Immune Microenvironment of GAC

**Figure 5:**
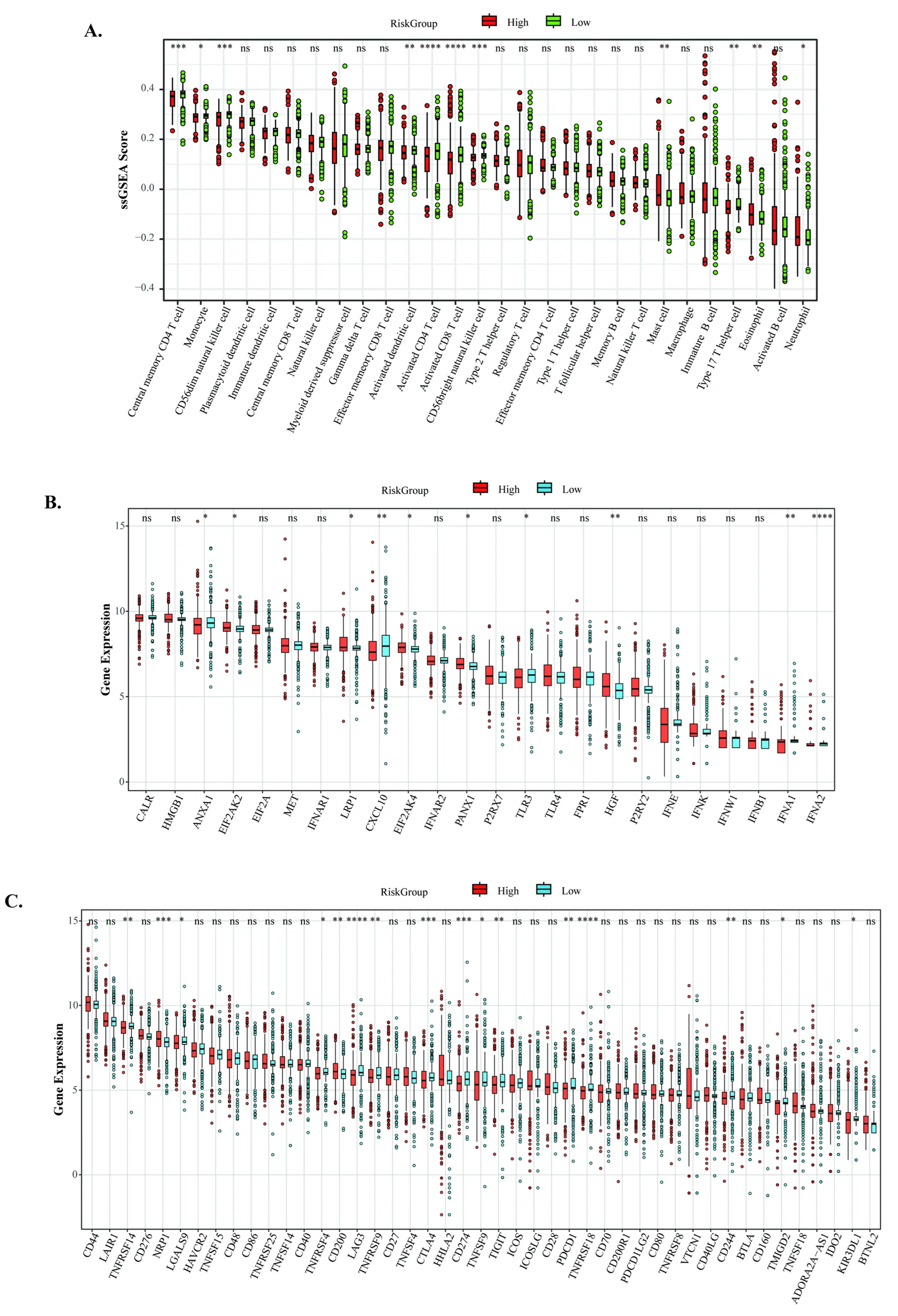
Immune Microenvironment Analysis of the GAC Neoantigen Prognosis Model. (**A**) Abundance of 28 immune cell types in high-risk and low-risk groups, stratified by the median risk score. (**B**,**C**) Expression levels of ICI and ICP-related genes in high-risk and low-risk groups. (* *p* < 0.05; ** *p* < 0.01; *** *p* < 0.001; **** *p* < 0.0001; ns indicates no statistical significance).

We analyzed the differences in TME of GAC patients. The results revealed that compared to normal tissues, the overall immune infiltration score in gastric cancer tissues was significantly lower (Figure S5A), suggesting a tendency toward overall immunosuppression in the tumor group. Importantly, the immune cell infiltration score was significantly higher in low-risk patients with favorable prognoses than in high-risk patients (Figure 5A), indicating that abundant immune cell infiltration is associated with better clinical outcomes.

We further analyzed the expression of immunogenic cell death (ICD) and immune checkpoint (ICP)-related genes in GAC patients. Regarding ICD, genes such as ANXA1, CXCL10, and TLR3 were more highly expressed in low-risk patients (Figure 5B), suggesting that GAC may help low-risk patients achieve favorable survival outcomes by modulating inflammatory responses and activating the type I interferon pathway. In contrast, genes such as EIF2AK2, EIF2AK4, HGF, and LRP1 were highly expressed in high-risk patients (Figure 5B). These genes may be involved in cellular stress adaptation, promoting invasion and metastasis, and regulating immunosuppression, thereby creating conditions conducive to malignant tumor progression and immune evasion.

Regarding ICP, inhibitory checkpoint genes such as PDCD1, CTLA-4, LAG3, and TIGIT were highly expressed in the low-risk patient group (Figure 5C). This phenomenon suggests that immunosuppressive signals may persist in the GAC microenvironment, potentially promoting gradual T-cell exhaustion and thereby driving the progression of low-risk patients toward a high-risk status. Simultaneously, significantly elevated expression levels of genes such as NRP1 and CD200 were observed in high-risk patients (Figure 5C). The above results indicate that the TME of GAC patients influences their survival prognosis. These findings reveal TME characteristics associated with prognostic risk stratification in GAC: the TME of the low-risk group exhibits stronger immune activation potential but also shows signs of exhaustion, whereas the TME of the high-risk group demonstrates more comprehensive immunosuppressive and pro-tumor progression features.

### 2.6. Clinical Significance of the GAC Neoantigen Prognostic Model

**Figure 6:**
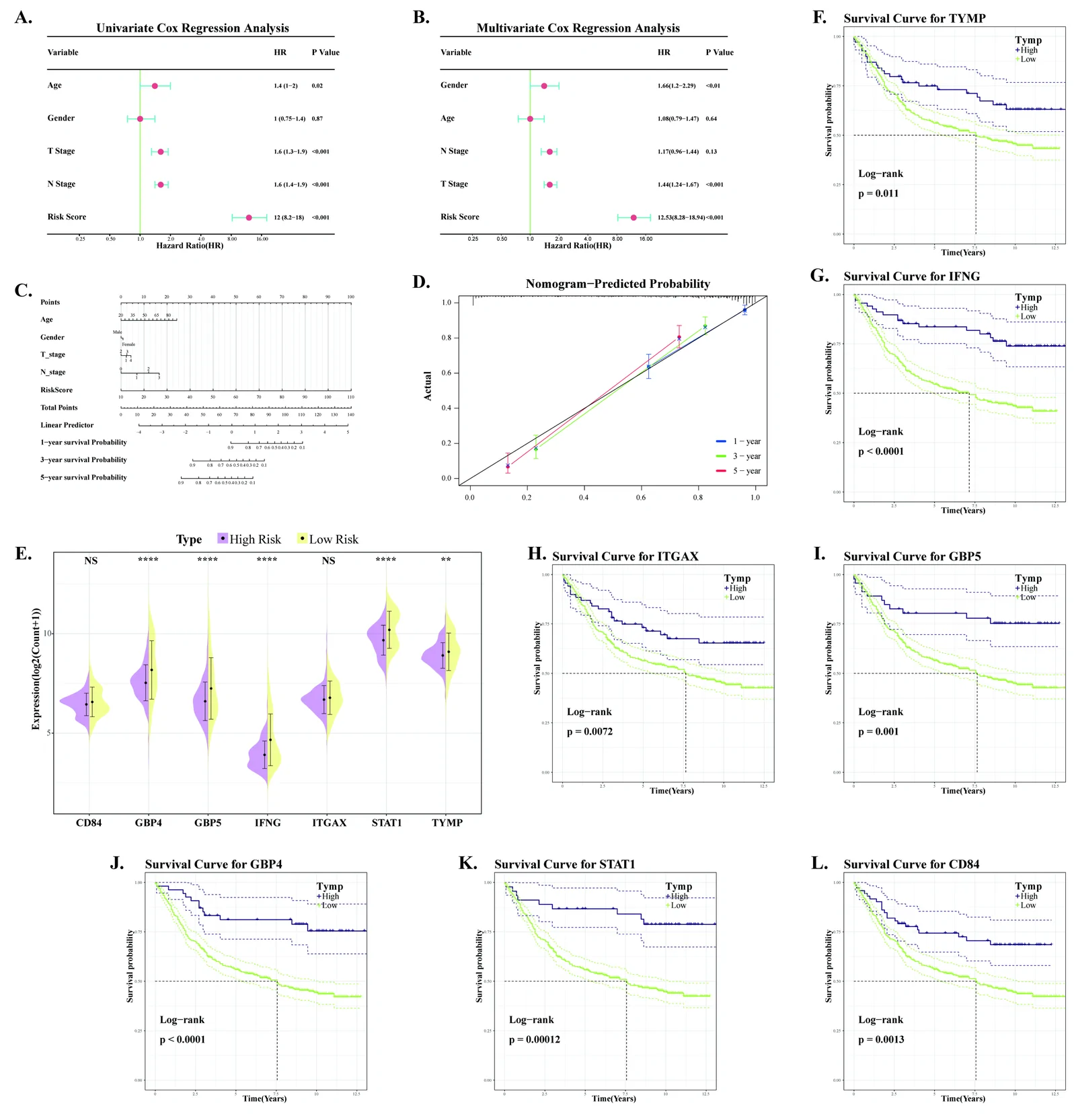
Clinical Performance Evaluation of the GAC Neoantigen Prognosis Model. (**A**) Univariate Cox regression analysis of clinical information in GAC. (**B**) Multivariate Cox regression analysis of clinical information in GAC. (**C**) Nomogram of the GAC risk model. (**D**) Calibration curve of the GAC risk model. (**E**) Expression differences in prognosis-related genes between high-risk and low-risk groups. (**F**–**L**) KM curves of prognosis-related genes with high and low expression in GAC. (* *p* < 0.05; ** *p* < 0.01; *** *p* < 0.001; **** *p* < 0.0001; NS indicates no statistical significance).

In the investigation of the clinical value of GAC, we performed univariate and multivariate Cox regression analyses. The univariate Cox analysis revealed that risk score, age, T stage, and N stage were associated with the prognosis of GAC patients; the risk score showed a correlation coefficient (*p* = 0.001, HR = 12, 95% CI: 8.2–18) (Figure 6A). Subsequently, multivariate Cox regression analysis was conducted, identifying age, T stage, and risk score as independent prognostic factors for GAC, with the risk score demonstrating a correlation coefficient (*p* = 0.01, HR = 12.53, 95% CI: 8.25–18.94) (Figure 6B). These findings suggest that the risk score derived from the GAC neoantigen prognostic model has favorable predictive efficacy.

We used a nomogram to visualize different clinical characteristics and risk scores of patients to predict their survival probabilities at 1, 3, and 5 years (Figure 6C). At the same time, the calibration curves for 1-, 3-, and 5-year survival rates demonstrated a high consistency between the actual and predicted survival rates (Figure 6D), indicating that the nomogram possesses strong clinical predictive value.

To analyze the expression of prognostic genes in cancer tissues, we categorized patients into high- and low-risk groups based on the median risk score and visualized the expression levels of the prognostic genes in these groups. The results showed that the expression of these genes was significantly higher in the low-risk patient group compared to the high-risk group (Figure 6E). Furthermore, Kaplan–Meier survival analysis indicated that high expression of TYMP, IFNG, ITGAX, GBP5, GBP4, STAT1, and CD84 was associated with better patient survival outcomes (Figure 6F–L). Therefore, these findings suggest that elevated expression of these prognostic genes may be beneficial for patients. Consistently, lower expression of these genes was observed in the high-risk group, which was correlated with worse survival.

### 2.7. Investigating the Role of Prognostic Genes

**Figure 7:**
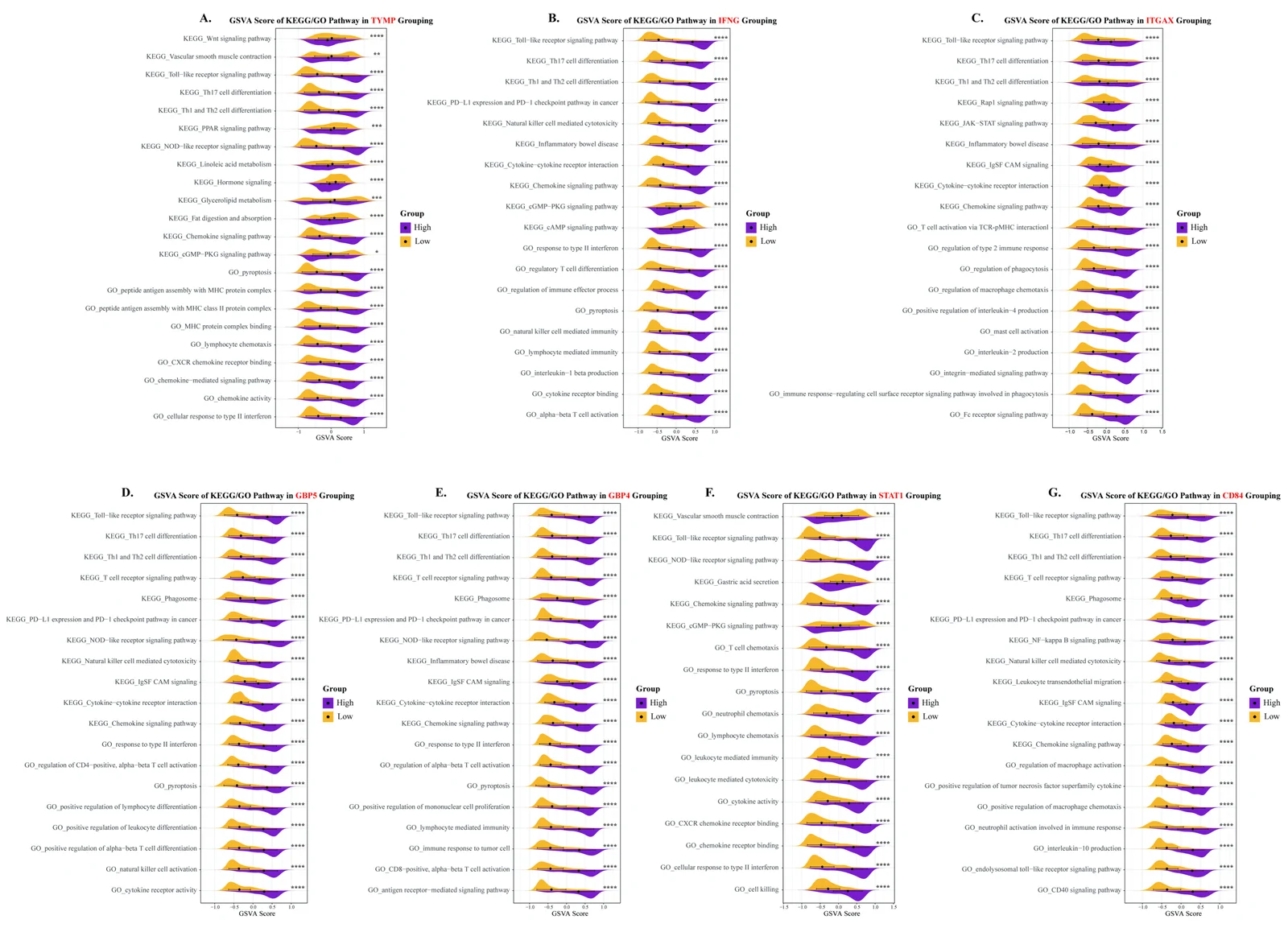
Exploring the Regulatory Mechanisms of Prognosis-Related Genes: GO and KEGG Pathway Enrichment Scores Covering Prognosis-Related Genes in GAC High-Risk and Low-Risk Groups. GSVA scores of GO and KEGG pathway enrichment for TYMP (**A**), IFNG (**B**), ITGAX (**C**), GBP5 (**D**), GBP4 (**E**), STAT1 (**F**), and CD84 (**G**) in high-risk and low-risk groups. (* *p* < 0.05; ** *p* < 0.01; *** *p* < 0.001; **** *p* < 0.0001).

To investigate the potential mechanisms of GAC prognosis-related genes in GAC, we divided the samples into high-expression and low-expression groups based on the median expression level of each gene and performed differential analysis (Figure S6A–G). Subsequently, we conducted GO functional and KEGG pathway enrichment analyses on the identified differentially expressed genes and quantified the activity scores of the core enriched pathways using GSVA. The GSVA results for TYMP showed upregulated activity in immune- and inflammation-related pathways as well as metabolic pathways, while cancer-related pathway activity was downregulated (Figure 7A). The GSVA results for IFNG indicated an upward trend in pathways related to GAC immune activation, immune regulation, inflammatory responses, and associated signal transduction (Figure 7B). The GSVA of ITGAX demonstrated significantly enhanced activity in pathways related to immune response and intercellular communication (Figure 7C). The GSVA results for GBP5 suggested upregulated activity in pathways such as tumor immunotherapy, infection, and immunity (Figure 7D). The GSVA of GBP4 revealed increased activity in pathways related to immunotherapy, regulation of the tumor immune microenvironment, and mechanisms of autoimmune diseases (Figure 7E). The GSVA results for STAT1 indicated upregulated activity in pathways associated with the immune system, cardiovascular function, and gastric secretion systems (Figure 7F). The GSVA of CD84 showed significantly enhanced activity in pathways related to immune cell interactions, immune regulation, and immunotherapy targets (Figure 7G).

The above results demonstrate that the high-expression groups of the seven prognostic genes collectively exhibited significant upregulation in a series of pathway activities, predominantly concentrated in core biological processes such as immune response, inflammatory reactions, and intercellular communication. This suggests a close association between the high expression of these genes and favorable patient prognosis. Notably, the PD-1/PD-L1 checkpoint pathway was also upregulated in the high-expression groups of multiple genes, indicating that compensatory immunosuppressive mechanisms may exist alongside immune activation. In summary, these GAC prognostic genes may play a critical role in GAC progression, immune evasion, and even treatment response by coordinately regulating immune-related signaling pathways and influencing TME and intercellular communication. These findings provide a theoretical foundation and potential directions for subsequent targeted interventions.

### 2.8. Molecular Docking and Molecular Dynamics Simulations

**Figure 8:**
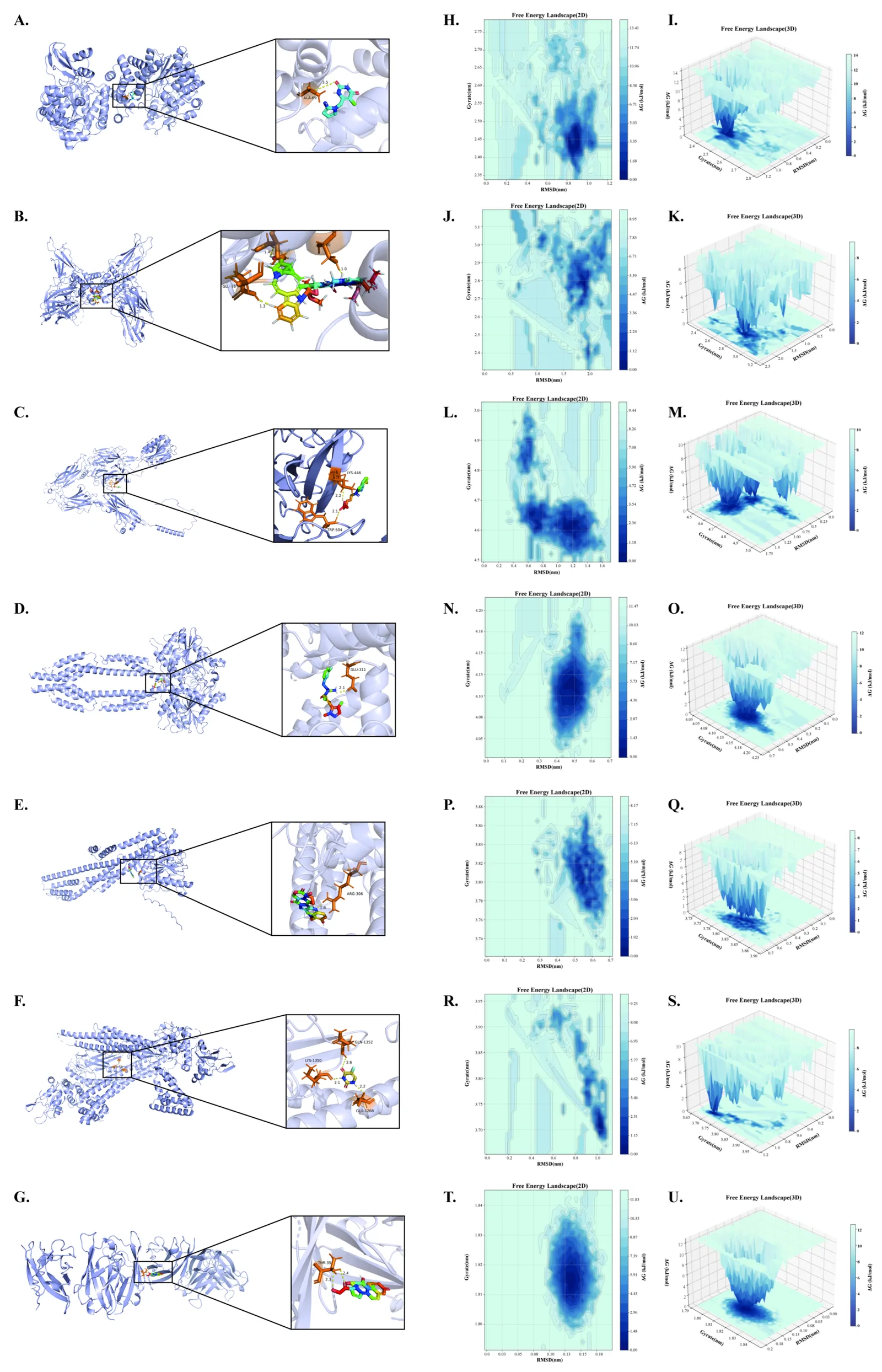
Binding Results of Prognosis-Related Gene-Encoded Proteins with Small Molecule Compounds and Molecular Dynamics Simulation Validation. (**A**) Docking result of TYMP with 5-chloro-6-uracil hydrochloride. (**B**) Docking result of IFNG with vincristine. (**C**) Binding result of ITGAX with 9-cis-retinoic acid. (**D**) Docking result of GBP5 with Afuresertib. (**E**) Docking result of GBP4 with topotecan. (**F**) Docking result of 5-fluorouracil with 5-fluorouracil. (**G**) Docking result of CD84 with prednisolone. (**H**,**J**,**L**,**N**,**P**,**R**,**T**) 2D free energy landscape maps of molecular dynamics simulations for prognosis-related genes and corresponding drugs. (**I**,**K**,**M**,**O**,**Q**,**S**,**U**) 3D free energy landscape maps of molecular dynamics simulations for prognosis-related genes and corresponding drugs.

To guide these drugs toward a therapeutic direction beneficial for patients, we utilized CTD to screen for targeted drugs and employed molecular docking techniques to identify drugs targeting the prognostic genes. Subsequently, molecular dynamics simulations were performed based on the binding outcomes between the drugs and their encoded proteins to verify the stability of their binding sites. Based on molecular docking simulations, we found that 5-chloro-6-uracil hydrochloride binds tightly to TYMP (Figure 8A) with a binding energy of −4.6 kcal/mol. Vincristine binds tightly to IFNG (Figure 8B) with a binding energy of −28.75 kcal/mol. 9-cis retinoic acid binds tightly to ITGAX (Figure 8C) with a binding energy of −6.94 kcal/mol. Afuresertib binds tightly to GBP5 (Figure 8D) with a binding energy of −3.92 kcal/mol. Topotecan binds tightly to GBP4 (Figure 8E) with a binding energy of −11.54 kcal/mol. 5-fluorouracil binds tightly to STAT1 (Figure 8F) with a binding energy of −2.99 kcal/mol. Prednisolone binds tightly to CD84 (Figure 8G) with a binding energy of −7.1 kcal/mol.

Gibbs free energy landscape diagrams characterize the conformational changes and rotations of proteins by analyzing their RMSD and RMSF, reflecting the stability of the complex. We successfully constructed three-dimensional free energy binding landscapes for six prognostic genes, with Gibbs free energy represented on the z-axis (Figure 8I,K,M,O,Q,S,U). The free energy landscapes effectively illustrate the energetic characteristics and conformational transition pathways of ligand-receptor interactions. The dark blue regions indicate low-energy conformational states, representing stable configurations. The low-energy conformational region of TYMP exhibits an RMSD of approximately 0.8–1.0 and an RMSF of about 2.4–2.5 (Figure 8H). The low-energy conformational region of IFNG shows an RMSD of approximately 1.55–2.0 and an RMSF of about 2.7–2.9 (Figure 8J). The low-energy conformational region of ITGAX displays an RMSD of approximately 1.0–1.4 and an RMSF of about 4.55–4.65 (Figure 8L). The low-energy conformational region of GBP5 has an RMSD of approximately 0.4–0.55 and an RMSF of about 4.1–4.13 (Figure 8N). The low-energy conformational region of GBP4 shows an RMSD of approximately 0.5–0.6 and an RMSF of about 3.78–3.84 (Figure 8P). The low-energy conformational region of STAT1 exhibits an RMSD of approximately 0.85–1.2 and an RMSF of about 3.68–3.73 (Figure 8R). The low-energy conformational region of CD84 displays an RMSD of approximately 0.13–0.15 and an RMSF of about 1.8–1.82 (Figure 8T). The Gibbs free energy landscape analysis combined with RMSD and RMSF measurements revealed that all protein–drug complexes formed distinct low-energy conformational regions, indicating favorable conformational stability of the systems during molecular dynamics simulations. The dark blue low-energy zones correspond to stable conformational states, suggesting that ligand binding significantly restricts both global and local conformational fluctuations of the proteins. Among them, the CD84 complex exhibited the lowest RMSD and RMSF values, demonstrating the strongest conformational stability, whereas IFNG and ITGAX maintained low free-energy states while allowing a certain degree of conformational flexibility, reflecting the dynamic adaptability of protein–ligand interactions. Overall, these results indicate that the interactions between the drugs and the seven key target proteins exhibit stable and ordered binding patterns. The seven identified small-molecule compounds may possess the potential to modulate adverse prognoses associated with prognostic genes, thereby offering new avenues for further research into adjuvant therapies for GAC.

## 3. Discussion

GAC is not only the most prevalent form of gastric cancer but also one of the most widespread malignancies worldwide [3]. Current treatment options for GAC remain limited, primarily relying on surgery and chemotherapy, underscoring the urgent need for additional prognostic and therapeutic strategies [4]. mRNA vaccines represent a powerful form of cancer immunotherapy due to their high efficacy, specificity, versatility, rapid development, and scalability [8,9], with neoantigens serving as ideal targets for tumor mRNA vaccines [14]. In this study, we systematically analyzed commonly mutated genes associated with cancer immunity in GAC using multi-omics data. Our results demonstrated that these genes not only harbor missense mutations but are also associated with antigen presentation and MHC class II molecules in GAC. Furthermore, pathway enrichment analysis revealed their regulatory roles in human immune responses.

In this study, we compared the differences between gastric gland epithelial cell populations and cancer cell populations using cancer hallmark genes [15,16,17]. We observed that the antigen presentation score of the cancer cell population was slightly higher than that of the gastric gland epithelial cell population. We therefore hypothesize that tumor cells may attenuate the immune clearance effect mediated by antigen presentation through certain immune escape mechanisms [18]. These results validate the accurate segregation of GAC cancer cell populations in our study and reveal the unique regulatory characteristics of cancer cells in signaling pathways, highlighting the complexity of the regulatory mechanisms underlying GAC.

In this study, we compared the molecular characteristic differences between gastric glandular epithelial cell populations and cancer cell populations based on single-cell transcriptome data. Pseudotime analysis results showed that epithelial cells were located at the early stage of differentiation, whereas cancer cells clustered at the terminal differentiation stage, suggesting that the transformation from gastric glandular epithelial cells to cancer cells is a gradual process accompanied by continuous transcriptional regulatory alterations. GSEA results further revealed that the epithelial cell stage was primarily characterized by activation of the TNF signaling pathway, indicating that cells are under long-term inflammatory stimulation. Chronic inflammation is considered an important causative factor in gastric carcinogenesis, as persistent inflammatory stimulation can induce DNA damage, promote cell proliferation, and increase the risk of mutation accumulation, thereby progressively driving epithelial cells toward malignant transformation [19]. Consistent with this, we observed that C > T mutations were the predominant mutation type in SNV analysis. This mutation pattern is typically associated with inflammation-related deamination reactions and oxidative stress damage, suggesting that the chronic inflammatory microenvironment may play a crucial role in GAC mutation formation [20,21]. Therefore, our study indicates that GAC development may undergo a gradual evolutionary process of “inflammation-driven epithelial cell alterations—mutation accumulation—immune regulation imbalance”. While retaining some immune-related functions, cancer cells achieve immune evasion and sustained proliferation through regulation of key signaling pathways [22]. This not only validates our accurate identification of cancer cell populations but also further reveals the complexity of interactions among inflammatory responses, gene mutations, and tumor immune regulation during GAC development and progression.

Upon entering host cells, neoantigen mRNA vaccines rely on the endogenous transcriptional and translational machinery to synthesize antigen proteins, which subsequently activate immune responses through antigen processing and presentation pathways [23]. To screen for potential neoantigen candidates, we performed an intersection analysis of upregulated genes, SNV-mutated genes, CNV-altered genes, and tumor immunity-related genes in GAC, ultimately identifying 10 candidate genes. A total of 101 machine learning algorithms were employed to optimize the construction of a prognostic model. Within the best-performing model interval, we applied RSF to develop a prognostic risk model, which identified TYMP, IFNG, ITGAX, GBP5, GBP4, STAT1, and CD84 as key prognosis-related genes. TYMP is a key enzyme involved in pyrimidine nucleoside metabolism. In GC, TYMP promotes DNA synthesis by regulating thymidine metabolism and is also involved in tumor angiogenesis, thereby supplying tumors with essential nutrients and oxygen for growth [24]. IFNG is a key immunoregulatory cytokine secreted by activated T cells and natural killer cells, playing a central role in antitumor immune responses [25]. In GC, IFN-γ upregulates the expression of MHC molecules and antigen processing-related components via activation of the JAK/STAT signaling pathway, thereby enhancing tumor antigen presentation and promoting the recognition and cytotoxic function of CD8+ T cells [26]. ITGAX is a surface receptor molecule predominantly expressed on immune cells, particularly DCs and a subset of macrophages. In GC, ITGAX promotes the proliferation, migration, invasion, and in vivo tumorigenic capacity of gastric cancer cells through the epithelial–mesenchymal transition pathway [27]. GBP5 and GBP4 encode members of the interferon-stimulated gene family. In GC, GBP5 and GBP4 can be induced by IFN-γ and participate in antigen presentation and inflammatory responses through the regulation of immune signaling pathways [28]. STAT1 is a core downstream molecule of the IFN-γ signaling pathway. In gastric cancer, STAT1 is activated by IFN-γ and translocates to the nucleus, where it regulates the expression of interferon-stimulated genes and MHC molecules, thereby enhancing tumor antigen presentation and cytotoxic T cell-mediated immune killing [29]. Furthermore, STAT1 is also involved in regulating inflammatory responses and the formation of TME, playing a significant role in immune surveillance and tumor progression in GC [30]. CD84 is an immunoregulatory receptor primarily expressed on the surface of T cells, B cells, and DCs. CD84 is expressed on various immune cell types and promotes immune evasion by enhancing the regulation of B cell function, promoting the expansion of myeloid-derived suppressor cells, and upregulating immune checkpoint molecules such as PD-L1 [31]. Our findings revealed that these prognostic genes were positively correlated with the immune infiltration of antigen-presenting cells and were also associated with the expression of MHC-related molecules, suggesting that these five prognostic genes may play a crucial role in activating antigen presentation responses.

This study revealed a strong association between immune characteristics and prognostic risk through a stratified analysis of TME in high-risk and low-risk groups of GAC patients. The high-risk group was enriched for myeloid immune cells, including macrophages and neutrophils, accompanied by high expression of IFN family cytokines, suggesting the presence of an immunosuppressive microenvironment characterized by inflammatory responses. These myeloid cells may promote tumor progression through the secretion of immunosuppressive factors and the induction of T cell exhaustion, while the elevated expression of IFN family cytokines may represent a compensatory response of tumor cells to immune pressure [32,33]. Concurrently, the high expression of EIF2AK2, EIF2AK4, HGF, and LRP1 in the high-risk group reflects the capacity of tumor cells to drive malignant progression through multiple mechanisms involving stress adaptation, invasion and metastasis, and immunosuppression [34,35]. The low-risk group was enriched for cytotoxic immune cells, including CD8^+^T cells and natural killer cells, along with high expression of chemokines such as CXCL10, indicating a more active antitumor immune response. CXCL10 recruits effector T cells to infiltrate tumor tissues, and together with the cytotoxic activity of natural killer cells, synergistically suppresses tumor growth [36]. Furthermore, the low-risk group exhibited high expression of ICD-related genes including ANXA1, CXCL10, and TLR3, suggesting the presence of active inflammatory responses and activation of the type I interferon pathway. The synergistic effects of these pathways may represent a core mechanism underlying the favorable survival prognosis observed in this patient subgroup [37]. At the immune checkpoint level, the high expression of inhibitory molecules including PDCD1, CTLA-4, LAG3, and TIGIT in the low-risk group reveals that even in patients with favorable prognosis, persistent immunosuppressive signals remain within the TME, suggesting that antitumor immune responses exist in a dynamic balance between “activation and exhaustion.” Prolonged immune checkpoint signaling may drive the progression of patients toward a high-risk state [38]. In contrast, the elevated expression of NRP1 and CD200 in the high-risk group further potentiates immunosuppressive characteristics, collectively forming a more comprehensive immunosuppressive network [39]. We therefore propose that the heterogeneity of the TME in GAC patients is closely associated with prognostic risk stratification: the low-risk group TME exhibits greater immune activation potential, yet may harbor early warning signs of T cell exhaustion; whereas the high-risk group TME displays synergistic features of “immunosuppression coupled with tumor promotion.”

This study revealed that TYMP, IFNG, ITGAX, GBP5, GBP4, STAT1, and CD84 were highly expressed in low-risk group patients, and their elevated expression was significantly associated with prolonged overall survival. This finding, which at first glance appears to contradict the conventional notion that high expression of oncogenes predicts poor prognosis, may reflect the protective role of an immune-active TME in GAC. IFNG is a core cytokine mediating both type I and type II immune responses, and its high expression typically signifies the activated status of effector T cells and NK cells [25]. STAT1, as a key transcription factor downstream of IFNG signaling, cooperates with IFNG to drive Th1-type immune responses [30]. ITGAX serves as a DC marker, and its high expression indicates abundant infiltration of antigen-presenting cells [27]. CD84 is an immunoregulatory receptor of the SLAM family, participates in T cell–antigen-presenting cell interactions and immune synapse formation [31]. GBP4 and GBP5 are members of the interferon-inducible GTPase family; recent studies have demonstrated their multifaceted roles in antitumor immunity, including promoting inflammasome activation, enhancing antigen presentation, and suppressing tumor metastasis [28]. Although TYMP has traditionally been regarded as a pro-angiogenic factor, in the context of TME, its expression has also been associated with macrophage polarization and chemokine secretion, potentially exerting indirect protective effects through the recruitment of immune cells [24]. Collectively, these seven genes constitute a highly synergistic antitumor immune response network, and their high expression profile suggests the presence of active antigen recognition, effector cell infiltration, and immune-mediated killing within the tumor microenvironment. This interpretation is further supported by our GSVA results, which demonstrated that groups with high expression of each of the seven genes were significantly enriched in pathways related to immune response, inflammatory response, cytokine signaling, and lymphocyte activation. Therefore, the essence of this gene signature lies in its representation of an immunologically “hot” tumor phenotype.

Targeted drug screening has emerged as a novel strategy for disease treatment [40]. Employing this approach, we performed targeted drug screening for the prognostic genes to explore alternative therapeutic options. 5-Chloro-6-uracil hydrochloride is a uracil derivative belonging to the pyrimidine class of compounds. This compound is not a conventional clinical antitumor agent; rather, it is primarily utilized as a biochemical intervention molecule in nucleic acid metabolism and enzymology research [41]. Vincristine, a classic chemotherapeutic agent derived from plants, has served as a cornerstone of combination chemotherapy regimens and has maintained a prominent role in the treatment of hematologic malignancies for decades [42]. However, it is not a mainstream option in gastric cancer therapy. Furthermore, its dose-limiting toxicity is peripheral neuropathy, which exhibits a cumulative, dose-dependent exacerbation pattern and requires close clinical monitoring [43]. 9-cis-Retinoic Acid is a retinoic acid receptor agonist belonging to the retinoid class of compounds. This compound is not a widely used conventional antitumor chemotherapeutic agent; rather, it is primarily employed as a cell differentiation inducer and a signaling pathway modulator in targeted therapy research. Due to its ability to activate both retinoic acid receptors and retinoid X receptors, it regulates the transcription of genes involved in cell proliferation, differentiation, and apoptosis, demonstrating potential in experimental studies to induce tumor cell differentiation and inhibit proliferation [44]. Afuresertib is an oral, selective AKT kinase inhibitor belonging to the class of small-molecule targeted therapeutic agents. This compound is not a widely marketed conventional antitumor drug; rather, it remains primarily in the clinical research phase as a targeted intervention molecule for tumors characterized by aberrant activation of the PI3K/AKT/mTOR signaling pathway. By reversibly binding to and inhibiting the activity of AKT1/2/3, it blocks downstream pro-survival and proliferative signaling, thereby inducing tumor cell apoptosis and suppressing tumor growth [45]. Topotecan is a semi-synthetic camptothecin derivative and a topoisomerase I inhibitor chemotherapeutic agent. This compound is an established conventional antitumor drug that, through specific inhibition of the religation function of topoisomerase I, induces irreversible DNA single-strand breaks, thereby blocking DNA replication and triggering tumor cell apoptosis. Topotecan is a well-validated standard treatment option in small cell lung cancer, ovarian cancer, and cervical cancer, commonly employed as second-line or later-line therapy [46,47]. 5-Fluorouracil is a uracil analog and a cornerstone of antimetabolite chemotherapeutic agents. This compound is one of the most widely used and historically established conventional antitumor drugs in clinical practice. It interferes with nucleic acid metabolism through multiple mechanisms: following intracellular conversion into active metabolites, it inhibits thymidylate synthase, thereby blocking the production of deoxythymidine monophosphate; simultaneously, its metabolites can be incorporated into RNA and DNA, disrupting their normal function and synthesis, ultimately leading to tumor cell death [48]. In the treatment of numerous solid tumors, including colorectal cancer, gastric cancer, esophageal cancer, breast cancer, head and neck squamous cell carcinoma, and pancreatic cancer, 5-fluorouracil serves as the backbone of most combination chemotherapy regimens, frequently administered in conjunction with oxaliplatin, irinotecan, leucovorin, or targeted agents [49]. Prednisolone is a synthetic glucocorticoid belonging to the class of steroidal anti-inflammatory and immunosuppressive agents. As one of the most essential hormonal drugs in clinical practice, it exerts broad and potent anti-inflammatory and immunosuppressive effects, modulates metabolism, and induces lymphocyte apoptosis, thereby playing a critical supportive and management role in oncological therapy [50]. Currently, prednisolone is primarily used in the treatment of prostate cancer [51]. Based on the targeted drug data corresponding to the seven prognostic genes identified, this approach may help mitigate the adverse prognosis associated with these genes and improve patient survival.

The innovation of this study lies in the precise identification of malignant cell populations in GAC patients through the analysis of large-scale single-cell RNA sequencing datasets. Furthermore, by integrating additional multi-omics data, we accurately delineated the characteristic mutation-associated gene set specific to GAC cancer cells. We employed ten machine learning methods to generate 101 algorithmic combinations to screen the scope of prognostic models and selected RSF within the optimal range to construct the prognostic model. Subsequently, we analyzed the correlations between the prognostic genes and the immune infiltration of antigen-presenting cells as well as MHC class II molecules to evaluate the antigen presentation capacity of the model, and we designed corresponding vaccine sequences using vaccine design platforms. Another innovative aspect of this study is the exploration of the functional roles of each prognostic gene and the investigation of targeted drug strategies, thereby offering multiple auxiliary therapeutic options for GAC.

Nevertheless, this study has several limitations. We identified relevant studies on these seven prognostic genes in GAC primarily through literature review. Additionally, the limited drug information available in the CTD database may have led to the omission of higher-quality or more precisely targeted agents. Beyond the above limitations, the AI-driven immunotherapeutic and vaccine design strategies adopted in this study also entail inherent biological and safety risks that cannot be overlooked, which are common challenges for current AI-assisted tumor immunotherapy development.

AI-optimized protein therapeutics and de novo antigens may contain cryptic epitopes that trigger unexpected anti-drug immune responses and neutralize therapeutic efficacy. Existing computational immunogenicity prediction tools have limited accuracy across diverse HLA alleles and fail to fully account for post-translational modifications and conformational characteristics, making them unable to precisely evaluate in vivo immunogenicity [52]. AI-predicted protein structures suffer from unstable reliability. Deep learning models exhibit poor predictive performance for artificially engineered protein sequences deviating from natural evolutionary patterns, and high-confidence structural predictions cannot guarantee correct protein folding and thermodynamic stability. Misfolded and aggregated proteins further aggravate immunogenic risks and impair therapeutic function [53]. Current AI prediction models are probabilistic and cannot completely simulate the complex co-evolution relationship between tumor clones and the host immune system. Tumor immunoediting and mutations in antigen presentation pathways may lead to antigen loss and immune escape, ultimately resulting in the failure of AI-designed vaccines and immunotherapies [54]. To mitigate the above risks, multi-layered verification and optimization strategies are required, including comprehensive in silico immunogenicity screening combined with in vitro immune functional verification, mandatory experimental validation of AI-predicted protein folding, stability and aggregation characteristics, and longitudinal monitoring of tumor antigen evolution to develop multi-epitope targeted therapeutic schemes. Fundamentally, AI serves only as an efficient hypothesis-generation and auxiliary design tool, which must be strictly verified by preclinical experiments and safety assessments rather than directly applied to clinical translation.

In future studies, we plan to integrate additional public drug database resources to identify more appropriate molecular candidates for targeted therapy. Meanwhile, we will conduct systematic experimental validation of the AI-designed GAC vaccine sequences and targeted therapeutic schemes in subsequent in vitro and in vivo experiments, and optimize the design strategy by monitoring tumor immune escape and immunogenic safety risks, so as to improve the clinical translational value of the prognostic model and supporting therapeutic strategies constructed in this study.

## 4. Materials and Methods

### 4.1. Data Sources for Analysis

In this study, we obtained scRNA-seq data for GAC from the Gene Expression Omnibus (GEO, https://www.ncbi.nlm.nih.gov/geo/, accessed on 25 August 2026) [55] and the National Genomics Data Center (NGDC, https://ngdc.cncb.ac.cn/omix/, accessed on 25 August 2026) [56]. The dataset includes a total of 497,559 cells from 90 patients: GSE183904 (26 patients), GSE268238 (38 patients), and OMIX001073 (26 patients). The scRNA-seq data were integrated and batch-corrected using Harmony (v1.2.1).

We downloaded bulk RNA sequencing (bulk RNA-seq) data for GAC from The Cancer Genome Atlas (TCGA, https://portal.gdc.cancer.gov/, accessed on 25 August 2026) [57] and GEO. From TCGA, we obtained data from 375 clinical tumor samples and 32 normal samples. From GEO, we acquired 433 clinical tumor samples from GSE84437, as well as 281 clinical samples and 89 adjacent non-tumor samples from GSE66229. The bulk RNA-seq data were integrated and batch-corrected using the limma package (v3.58.1).

The SNV dataset for GAC was obtained from TCGA. Pathway datasets used for scoring and immune-related genes were sourced from the Gene Set Enrichment Analysis (GSEA) database (https://www.gsea-msigdb.org/gsea/index.jsp, accessed on 25 August 2026) [58]. Our cancer immune-related gene sets were derived from the ImmPort Immunology Database (https://www.immport.org, accessed on 25 August 2026) [59].

The scRNA-seq data were used to accurately identify gastric gland epithelial cell group in GAC, SNV data were used to screen for mutated genes in GAC, and bulk RNA-seq data were employed to identify highly expressed genes in cancer tissues. Immune-related gene sets from the ImmPort database were utilized to select cancer immune-associated genes. For the construction of the subsequent neoantigen prognostic model, GAC patient samples from the TCGA and GSE66229 datasets were merged and then split into a 7:3 ratio to serve as the training set and internal validation set, respectively, while the GSE84437 dataset was used as an external validation set for the prognostic model.

### 4.2. Single-Cell Sequencing Analysis

First, we performed quality control (QC) on the scRNA-seq data using the following specific criteria: cells with nFeature_RNA between 500 and 6000 were retained; cells with nCount_RNA between 500 and 10,000 were retained; and cells with percent.mt ≥ 10% were excluded. After QC, we ultimately obtained 273,924 cells. Subsequently, we used Seurat (v4.4.0) for normalization, dimensionality reduction, and clustering of the scRNA-seq data at 12 principal components. Next, we selected cell subpopulations clustered at the 6th resolution level, performed automated annotation with SingleR (v2.4.1), and further refined the annotations manually with reference to the CellMarker database (http://117.50.127.228/CellMarker/, accessed on 25 August 2026) [60]. To validate the accuracy of the annotations, we visualized the expression of marker genes across different cell clusters using bubble plots.

### 4.3. Aneuploid Cell Clusters in GAC Were Defined as GAC Cancer Cell Populations

We performed CNV analysis on Gastric gland epithelial cell clusters using CopyKAT (v1.1.0) and defined those exhibiting aneuploidy features as cancer cells. Subsequently, we validated the accuracy of the CopyKAT-based cell classification by comparing the expression profiles of classic mutated genes and normal genes in GAC. Following this, we employed AUCell (v1.24.0) to assess differences in pathway activity related to antigen presentation between cancer cells and normal Gastric gland epithelial cells.

### 4.4. Pseudotime and Somatic Mutation Analysis

To analyze mutational patterns along the developmental trajectory of GAC cancer cells, we defined characteristic genes of GAC cancer cells as trajectory selection markers. Using Monocle (v2.30.1), we constructed the growth and developmental trajectory illustrating the transition from normal to cancerous cells in GAC. Building on this, we further employed GSEA to evaluate pathway enrichment in cells at different states along the trajectory.

In this study, we analyzed SNV data from GAC patient samples in TCGA using maftools (v2.18.0) and visualized the top 30 most frequently mutated genes via waterfall plots. The missense mutation genes identified from this screening were subsequently used for further analysis.

### 4.5. Multi-Omics Cross-Screening of Key Genes for the Construction of a Neoantigen Prognostic Risk Model

We first performed differential expression analysis between tumor tissues and adjacent normal tissues using the TCGA and GSE66229 datasets, with screening criteria set at |logFC| > 1 and *p* < 0.05, to identify genes significantly upregulated in GAC tissues. Subsequently, these upregulated genes were intersected with CNV genes identified from scRNA-seq data analysis, SNV genes obtained from TCGA, and cancer-related immune gene sets from ImmPort. Through this multi-omics cross-screening approach, a set of potential neoantigen-related genes for GAC was ultimately determined.

First, in constructing the GAC neoantigen prognostic risk model, we employed ten machine learning algorithms, including Supervised Principal Component Analysis (SuperPC), Generalized Boosted Regression Model (GBM), Survival Support Vector Machine (Survival-SVM), Elastic Net (Enet), Cox Partial Least Squares Regression (plsRcox), Least Absolute Shrinkage and Selection Operator (LASSO), Ridge Regression (Ridge), Stepwise Cox Regression (StepCox), CoxBoost, and RSF, resulting in a total of 101 algorithmic combinations. Subsequently, we evaluated the concordance index (C-index) of each model in both the training and validation sets, along with its average C-index, to comprehensively compare the robustness and predictive performance of the models [61,62]. Ultimately, we selected RSF from the range of top-performing models as the method to construct the prognostic risk model. RSF is capable of handling high-dimensional feature data while effectively capturing complex nonlinear relationships. Additionally, it is suitable for processing survival data with censored features and provides stable and accurate risk prediction [63]. Finally, we validated the model performance using Receiver Operating Characteristic (ROC) curves and survival analysis between high- and low-risk groups. Additionally, the independent predictive ability of this prognostic risk model in clinical practice was assessed through nomogram analysis, along with univariate and multivariate Cox regression analyses.

### 4.6. Antigen Presentation Efficacy of the GAC Prognostic Model

To evaluate whether GAC prognostic genes possess the potential to trigger antigen presentation in vivo, we assessed the correlation between these prognostic genes and APCs, specifically macrophages and DCs, using the Tumor Immune Estimation Resource (TIMER, https://cistrome.shinyapps.io/timer/, accessed on 25 August 2026) [64]. Protein–protein interaction information between prognostic genes and MHC-related pathway genes was obtained from the STRING database (https://cn.string-db.org/, accessed on 25 August 2026) [65] and visualized using Cytoscape (v3.10.3), with nodes ranked by degree to analyze potential functional associations between prognostic genes and MHC-related molecules. Immunohistochemistry images of prognostic genes in normal and cancer tissues were downloaded from the Human Protein Atlas database (HPA, https://www.proteinatlas.org/, accessed on 25 August 2026) [66] to compare their expression differences during tumorigenesis. Additionally, pathway enrichment analysis was performed on GAC patient data to observe pathway activity patterns in GAC patients. We utilized the vaccine design platform mRNAdesigner (https://www.biosino.org/mRNAdesigner/, accessed on 25 August 2026) [67] to design our mRNA templates.

### 4.7. The Immunological Microenvironment of GAC Patients

To assess the immune infiltration in the GAC prognostic risk model, we used the single-sample Gene Set Enrichment Analysis (ssGSEA) algorithm in the GSVA (v1.50.5) package to calculate the immune infiltration levels of 28 immune cell types, thereby investigating the association between high- and low-risk GAC patient groups and immune infiltration. Additionally, we compared the expression differences in immune checkpoint inhibitors (ICI) and immunogenic cell death (ICD)-related genes between the high- and low-risk patient groups.

### 4.8. Potential Functional Impact of Prognostic Genes on GAC Patients

To further investigate the potential impact of prognostic genes on GAC patients, we divided patients in the training set cancer group into high-expression and low-expression subgroups based on the expression levels of each prognostic gene. Specifically, patients ranked in the top 40% of expression were assigned to the high-expression group, while those ranked in the bottom 40% were assigned to the low-expression group. Subsequently, differential analysis was performed between the high- and low-expression groups, and the resulting gene sets were subjected to Gene Set Variation Analysis (GSVA) to assess changes in related pathway activities.

### 4.9. Small Molecule Drug Screening and Molecular Docking

To modulate the expression of prognostic genes in a manner beneficial for patient treatment, we used the therapeutic impact of prognostic genes from previous results as the screening criterion for drug candidates. First, we downloaded a library of small molecule compounds related to the prognostic genes from the Comparative Toxicogenomics Database (CTD, https://ctdbase.org/, accessed on 25 August 2026) [68] and obtained the structural information of these compounds from the PubChem database (https://pubchem.ncbi.nlm.nih.gov/, accessed on 25 August 2026) [69]. Subsequently, we retrieved and downloaded the biomolecular structures of proteins encoded by the prognostic genes from the UniProt database (https://www.uniprot.org/, accessed on 25 August 2026) [70]. We used AutoDock (Linux, v4.2) for molecular docking to explore the interactions between small molecule compounds and the proteins encoded by the prognostic genes. Following established screening criteria, automated docking was performed between the biomacromolecules and the small molecule compounds. During the docking process, significant interactions were determined based on the lowest binding energy. All docking results were visualized using PyMOL (v2.8, open-source version).

### 4.10. Molecular Dynamics Simulations to Validate Molecular Docking Results

Using GROMACS (v2020.6) software [71], we conducted MD simulations on the docked drug–protein complexes to assess the stability of the ligand binding sites. All systems were solvated in a cubic simulation box with explicit TIP3P water molecules. Prior to MD simulation, the docking outputs were preprocessed with AmberTools22: hydrogen atoms were added, and force field parameters were assigned for system construction. The protein was modeled using the Amber99sb-ildn force field, while small-molecule ligands were parameterized with the GAFF2 force field combined with AM1-BCC partial atomic charges. All protein-ligand systems underwent energy minimization via the steepest descent algorithm to eliminate steric clashes and optimize geometric conformations. Two sequential equilibration simulations were performed: NVT ensemble equilibration was conducted at 300 K with the V-rescale thermostat, followed by NpT ensemble equilibration at 1 bar using the Parrinello-Rahman barostat. Each equilibration phase was performed with a coupling constant of 0.1 ps over 100 ps. Finally, production MD simulations were carried out for a total duration of 100 ns under NpT ensemble conditions at 300 K and 1 bar. Upon completion of the MD simulations, key parameters of the complexes, including root-mean-square deviation (RMSD), root-mean-square fluctuation (RMSF), and Gibbs free energy profiles, were calculated using built-in GROMACS tools. The simulation results were visualized using Python (v3.11)-based plotting tools.

### 4.11. Statistical Methods and Associated Code

We performed all statistical analyses using R language (v4.3.2). Group differences were compared using nonparametric tests, and differences in survival probabilities between samples were assessed for significance by the log-rank test. In the statistical analysis, a significance threshold was set at *p* < 0.05 or FDR < 0.05. Correlations between variables were analyzed using the Spearman rank correlation method. All code used in this study has been publicly uploaded to the GitHub platform (https://github.com/qqxj/GAC, accessed on 25 August 2026).

## 5. Conclusions

This study is based on multi-omics data from GAC. Single-cell data were first utilized to identify cancer cells, followed by the integration of SNV and CNV mutation data, cancer-related immune gene sets, and upregulated genes in GAC. Using ten machine learning methods, 101 algorithm combinations were generated to construct a new antigen prognosis risk model. This model effectively predicts patient prognosis and immunotherapy efficacy. By analyzing the correlation between prognostic genes and APCs, as well as their protein interactions with MHC class II molecules, the potential role of these genes in antigen presentation was further validated. Additionally, we designed mRNA vaccines using the mRNA designer platform, revealed associations between prognostic genes and various adverse clinical factors, and elucidated through TME analysis how these genes regulate cancer cell proliferation, migration, and immune evasion to promote tumor progression. Furthermore, potential therapeutic drugs targeting these prognostic genes were identified, and their binding affinity to the targets was evaluated via molecular dynamics simulations, providing experimental evidence for targeted therapy. These findings offer a theoretical foundation for developing mRNA vaccines, personalized treatments, and novel targeted therapies for GAC patients.

## Data Availability

All the raw data in this article are from GEO database, TCGA database and OMIX database.

## Supplementary Materials

The following supporting information can be downloaded at: https://www.mdpi.com/article/10.3390/ijms27177712/s1.

## Abbreviations

The following abbreviations are used in this manuscript:

Gastric adenocarcinoma	GAC
Random Survival Forest	RSF
Molecular dynamics simulations	MD
Tumor microenvironment	TME
Copy number variation	CNV
Single nucleotide variation	SNV
Gastric cancer	GC
Dendritic cell	DC
Single-cell RNA sequencing	scRNA-seq
Antigen-presenting cells	APCs
Bulk RNA sequencing	bulk RNA-seq
Gene Set Enrichment Analysis	GSEA
Quality control	QC
Supervised Principal Component Analysis	SuperPC
Generalized Boosted Regression Model	GBM
Survival Support Vector Machine	Survival-SVM
Elastic Net	Enet
Cox Partial Least Squares Regression	plsRcox
Least Absolute Shrinkage and Selection Operator	LASSO
Ridge Regression	Ridge
Stepwise Cox Regression	StepCox
Concordance index	C-index
Receiver Operating Characteristic	ROC
Human Protein Atlas	HPA
Comparative Toxicogenomics Database	CTD
Root-mean-square deviation	RMSD
Root-mean-square fluctuation	RMSF
Overall survival	OS
Protein–protein interaction network	PPI
Immunogenic Cell Death	ICD
Immune Checkpoint	ICP
Major Histocompatibility Complex	MHC
Interferon-Stimulated Genes	ISGs
